# Inhibition of the PI3K/AKT/mTOR pathway activates autophagy and compensatory Ras/Raf/MEK/ERK signalling in prostate cancer

**DOI:** 10.18632/oncotarget.18082

**Published:** 2017-05-23

**Authors:** Dominika E. Butler, Christopher Marlein, Hannah F. Walker, Fiona M. Frame, Vincent M. Mann, Matthew S. Simms, Barry R. Davies, Anne T. Collins, Norman J. Maitland

**Affiliations:** ^1^ The Cancer Research Unit, Department of Biology, University of York, York YO10 5DD, UK; ^2^ Norwich Medical School, University of East Anglia, Norwich NR4 7TJ, UK; ^3^ Gastroenterology Research Department, Hull Royal Infirmary, Hull HU3 2JZ, UK; ^4^ Department of Urology, Castle Hill Hospital (Hull & East Yorkshire Hospital NHS Trust), Cottingham HU16 5JQ, UK; ^5^ Hull York Medical School, University of York, York YO10 5DD, UK; ^6^ AstraZeneca, Cancer Research UK Cambridge Institute, Cambridge CB2 0RE, UK

**Keywords:** prostate cancer, AKT, mTOR, RAS, MAPK signalling, autophagy

## Abstract

The PI3K/AKT/mTOR pathway is frequently activated in advanced prostate cancer, due to loss of the tumour suppressor PTEN, and is an important axis for drug development. We have assessed the molecular and functional consequences of pathway blockade by inhibiting AKT and mTOR kinases either in combination or as individual drug treatments. In established prostate cancer cell lines, a decrease in cell viability and in phospho-biomarker expression was observed. Although apoptosis was not induced, a G1 growth arrest was observed in PTEN null LNCaP cells, but not in BPH1 or PC3 cells. In contrast, when the AKT inhibitor AZD7328 was applied to patient-derived prostate cultures that retained expression of PTEN, activation of a compensatory Ras/MEK/ERK pathway was observed. Moreover, whilst autophagy was induced following treatment with AZD7328, cell viability was less affected in the patient-derived cultures than in cell lines. Surprisingly, treatment with a combination of both AZD7328 and two separate MEK1/2 inhibitors further enhanced phosphorylation of ERK1/2 in primary prostate cultures. However, it also induced irreversible growth arrest and senescence.

*Ex vivo* treatment of a patient-derived xenograft (PDX) of prostate cancer with a combination of AZD7328 and the mTOR inhibitor KU-0063794, significantly reduced tumour frequency upon re-engraftment of tumour cells.

The results demonstrate that single agent targeting of the PI3K/AKT/mTOR pathway triggers activation of the Ras/MEK/ERK compensatory pathway in near-patient samples. Therefore, blockade of one pathway is insufficient to treat prostate cancer in man.

## INTRODUCTION

Prostate cancer is the most common cancer in men, and the second most common cause of cancer death in men in the UK (Cancer Research UK, 2014). Advanced prostate tumours initially respond well to androgen deprivation therapy (ADT). However, in most cases, ADT ultimately fails and castration resistant prostate cancer (CRPC) emerges, which is almost inevitably fatal [1]. Although novel therapeutic agents, such as abiraterone and enzalutamide have demonstrated improved overall survival in metastatic CRPC, new therapies are urgently needed [2–4]. There is also a growing realisation that early adoption of chemotherapy either before or with hormone therapy, results in greatly increased survival [5].

The PI3K/AKT/mTOR pathway is over-activated in many human cancers, including prostate cancer, and has emerged as a target for small molecule therapies [6–8]. However, inhibition of the PI3K pathway alone has proven to be insufficient for treatment of cancer, due to the existence of feedback loops and cross-talk with other pathways, including the Ras/Raf/MEK/ERK [9–11], and androgen receptor signalling pathways [8, 12, 13]. Therefore, combined targeting of the PI3K/AKT/mTOR and the Ras/MEK/ERK pathways has been proposed as an alternative approach in a range of different cancers, including multiple myeloma (MM), melanoma and prostate cancer [6, 14, 15]. In multiple myeloma, combined blockade of the PI3K and MAPK pathways resulted in significantly enhanced cell death in the majority of tested cell lines [14]. A combination of the mTORC1 inhibitor, rapamycin and the MEK inhibitor, PD325901 inhibited growth of both androgen-responsive (CWR22Rv1 and CASP 2.1) and androgen-independent (CASP 1.1) prostate cancer cell lines. Blockade of both pathways, using a novel sorafenib derivative, NSK-01105, was shown to reduce cell proliferation and induce apoptosis in LNCaP and PC3 cells, and xenografted tumours of the same cell lines [16].

Preclinical assessment of potential new prostate cancer therapies is routinely performed in a limited number of cell lines and xenografts derived from them. The acknowledged heterogeneity between responses in different patients is therefore not addressed and may be the cause of the high attrition rate in oncology drug development [17]. In general, such approaches allow measurements of drug efficacy, but can prove misleading in determining the clinical applicability of drugs and drug combinations. Additionally, the presence of cancer stem cells (CSCs), in a range of cancers including leukaemia [18], breast [19], brain [20], colon [21], pancreas [22], lung [23] and prostate [24–26], has been suggested to be responsible for therapy resistance [27]. In prostate cancer, basal phenotype CSCs, CD44^+^/α_2_β_1_^hi^/CD133^+^ are the most likely source of treatment-resistant cells, serving as a reservoir for tumour recurrence after castration therapy [24, 28, 29].

To address intra-tumour heterogeneity and differ-ences between patients, we investigated the role of PI3K/AKT/mTOR signalling in a range of prostate cancer primary cultures and PDXs. By measuring the signalling and cellular responses after treatment with small molecule inhibitors of the pathway, we demonstrate a surprising, but consistent disparity between the ‘standard’ cell line models and patient-derived cells.

## RESULTS

### Novel AKT and mTOR inhibitors reduce prostate cell viability

As AKT and mTOR kinases are crucial regulators of prostate cancer cell growth, proliferation and survival, treatment of high grade tumours with an AKT kinase inhibitor (AZD7328) and mTOR kinase inhibitor (KU-0063794), both ATP-competitive small molecules, has been proposed as a promising treatment strategy in oncology [6–8]. To establish the efficacy of the small molecule inhibitors, two PTEN-positive cell lines, BPH1 (benign) and P4E6 (localised cancer) and two PTEN-negative cell lines LNCaP (lymph node metastasis) and PC3 (bone metastasis) were treated with a range of concentrations (0.01 – 30 μM) of AZD7328 or KU-0063794, for up to 48 hours. An MTS assay was performed to investigate the effect of AZD7328 and KU-0063794 on cell viability. LNCaP cells were the most sensitive to both inhibitors with an EC_50_ of 0.4 μM (0.3-0.5) for AZD7328 and 1.03 μM (0.8-1.3) for KU-0063794 (Table 1). PC3 cells were similarly susceptible to KU-0063794, but were 27 fold less sensitive to AZD7328 (11.0 μM). Although treatment with AZD7328 reduced the viability of both PTEN-negative cell lines, the PTEN-positive cells lines (BPH1 and P4E6) were less sensitive to the AKT inhibitor and their viability was only slightly reduced. Specifically, the EC_50_ for the KU-0063794 were approximately 5 – 15 fold higher for BPH1 (5.8 μM) and P4E6 (10.2 μM) in comparison to LNCaP and PC3 (Table 1). Taken together, these results suggest that AZD7328 and KU-0063794′s ability to reduce cell viability is dependent on PTEN status, with PTEN-negative cell lines being more susceptible to AKT and mTOR blockade.

To validate these findings in a ‘near-patient’ setting, primary cultures were generated from prostate cancer biopsies. Biopsies from patients with benign disease were used as normal controls. The cells were treated with increasing concentrations (1 – 50 γM) of AZD7328 or KU-0063794 for up to 72 hours and cell viability was assessed. The results show that AZD7328 and KU-0063794 are less effective in patient-derived cultures (Supplementary Figure 1) indicating that combined treatment should be considered.

**Figure 1:**
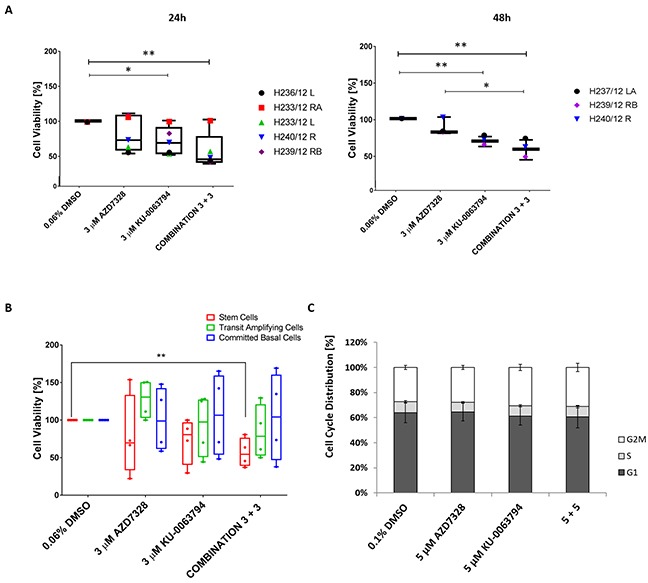
Cell viability decreases following inhibition of AKT and mTOR in patient-derived prostate cultures **(A)** Primary cultures derived from cancers (n=5) and normal prostate (n=1) were treated with either 3 μM AZD7328, 3 μM KU-0063794 or a combination of 3 μM AZD7328 + 3 μM KU-0063794 in triplicate for 24 hours and 48 hours. Following treatment, the cells were harvested, stained with trypan blue and counted. The percentage of viable cells was calculated and normalized to the vehicle control (0.04% DMSO). Significant differences (p value < 0.05) in cell viability are indicated on the graphs. **(B)** Primary prostate cancer samples H252/12, H163/12, H149/12, and H135/11 were treated with 3 μM AZD7328, 3 μM KU-0063297 or a combination of 3 μM AZD7328 + 3 μM KU-0063794 for 72 hours. Following treatment, the cells were harvested, sorted into committed basal (CB), transit amplifying (TA) and stem-like cells (SC) and counted using trypan blue exclusion. Percentage of viable cells was calculated and normalized relative to the vehicle control (0.06% DMSO). Error bars represent the standard deviation. Significant difference (p <0.05) in cell viability was only observed in stem cell fraction treated with the combination of 3 μM AZD7328 + 3 μM KU-0063794 in comparison to vehicle control (indicated on the graph). **(C)** Cell cycle distribution remains unchanged in primary prostate cultures after treatment with AZD7328 and KU-0063794. Five prostate cultures (2 BPH and 3 cancers) were treated with 5 μM AZD7328, 5 μM KU-0063794 or 5 μM AZD7328 + 5 μM KU-0063794 for 72 hours. Following treatment, non-adherent cells were removed and remaining cells were harvested, fixed with 70% ice-cold ethanol, and stained with propidium iodide and analysed by flow cytometry. Control cells were treated with 0.1% DMSO. The signal was recorded in the PE channel and debris (subG1 phase) were excluded from the analysis.

As mTOR kinase is responsible for maintaining metabolic homeostasis, the effect of treatment was measured using trypan blue exclusion to assess viability, and in a larger cohort of patients (n=6). Primary cultures were treated with AZD7328 and KU-0063794, either alone or in combination. We observed a significant decrease in the viability (∼ 30%) with 3 μM KU-0063794 treatment at 24 and at 48 hours (*P*< 0.015) (Figure 1A). Interestingly, a significant decrease in cell viability was observed in the majority of samples following combined treatment with 3 μM of either inhibitor at 24 hours and 48 hours (*P* < 0.006) (Figure 1A). Additionally, a significant advantage of combination treatment over AZD7328 alone was observed at 48 hours. Importantly, these data show that there is variability in response to treatment between patients.

Since prostate epithelial cells are organised in a hierarchy [24, 29–31], the effect of treatment was assessed on the viability of Stem-like Cells (SC), Transit Amplifying (TA) and Committed Basal (CB) cells. Primary epithelial cancer cultures (derived from patients with Gleason 9, n=3 and Gleason 7 cancer, n=1) were treated with either 3 μM AZD7328, 3 μM KU-0063794 or a combination of 3 μM AZD7328 + 3 μM KU-0063794, for 72 hours. The cell subpopulations were isolated following treatment, and viability was assessed using trypan blue exclusion. Treatment with 3 μM AZD7328 reduced the viability of stem-like cells in 3 of 4 samples with the highest reduction (78%), in a Gleason 9 sample (H149/12) (Figure 1B). Moreover, treatment with 3 μM KU-0063794 resulted in a decrease in the viability of SCs derived from all Gleason 9 samples, whilst stem cell numbers were not affected by treatment from the Gleason 7 culture. Interestingly, a combination of 3 μM AZD7328 + 3 μM KU-0063794 resulted in a significant decrease (*P* = 0.0044) in SC numbers in all cancer cultures (Figure 1B).

In contrast to the effect on stem cell numbers, TA and CB cell numbers were not affected with either inhibitor alone or a combination of both (Figure 1B). These results reflect prostate cancer intra tumoural heterogeneity as well as differences between patient samples further emphasizing the need for patient stratification.

### Inhibition of AKT and mTOR does not induce cell cycle arrest in primary cultures

To determine whether the inhibitors directly influenced the cell cycle, two BPH and three cancer cultures were treated for 72 hours, with 5 μM AZD7328, 5 μM KU-0063794, and a combination of 5 μM AZD7328 + 5 μM KU-0063794 and were subsequently analysed by flow cytometry. The results revealed no significant difference in cell cycle distribution (p value >0.3) (Figure 1C).

### Expression of the tumour suppressor PTEN in prostate cancer patient cultures

Loss of the tumour suppressor gene, PTEN is observed in 40% of advanced prostate cancers [32, 33]. Given the importance of PTEN in regulating the activity of the PI3K pathway, we determined the status of the PTEN gene as well as expression, in our patient-derived cultures. DNA analysis was performed on 7 samples for which matched lymphocytes and tumour biopsies were available. As a mutational hot spot has been reported in exon 5 of the PTEN gene (http://atlasgeneticsoncology.org/Genes/PTENID158.html), we focused our analysis on exon 5. The results showed that missense mutations were found in tumour DNA of three samples (H135/11, H224/12 and H278/13), but not in their corresponding lymphocytes (Supplementary Figure 2A). However, it is not clear whether those mutations have resulted in translation of a functional PTEN and be affecting functionality of the PTEN protein and this will need to be investigated further.

Next, we determined whether tumour cells showed loss of heterozygosity at the *PTEN* gene locus 10p23 using two LOH probes (Table 2) described in Dahia et al [34]. LOH analysis using Probe 1 (dinucleotide repeat in *PTEN* intron 2 (AFMa086wg9) showed that 2/7 samples (H135/11 and H377/13) were heterozygous and both cultures showed a relative loss of heterozygosity (expressed as reduction in peak intensity) in tumour DNA. Interestingly, the H135/11 sample which Had a missense mutation in exon 5 and LOH, showed a significant reduction in cell viability of stem-like cells following treatment with a combination of AZD7328 and KU-0063794, indicating higher susceptibility to the drugs. LOH analysis using probe 2 (G/T sequence polymorphism in *PTEN* intron 8) identified heterozygosity in a further 2 samples (H224/12 and H237/12). However, only H224/12 showed a relative loss of heterozygosity in the tumour DNA in comparison to the lymphocyte DNA.

**Table 2:** Primers used for PTEN mutational and LOH analysis

Primer sequences/analysis type	Forward	Reverse
**Exon 5 sequencing (mutational analysis)**	5′ ACCTGTTAAGTTTGTATGCAAC 3′	5′ TCCAGGAAGAGGAAAGGAAA 3′
**LOH intron 2 (AFMa086wg9)**	5′ AAATGTACGGTTCATTGACTT 3′	5′ GACTGACTACAAATGGGCA 3′
**LOH intron 8**	5′ CATTCTTCATACCAGGACCAG 3′	5′ TCATGTTACTGCTACGTAAAC 3′

Western blotting results showed that PTEN protein was detected in all primary cultures tested (Supplementary Figure 2B). The presence of PTEN in our cultures could possibly explain the limited efficacy of the AZD7328 and KU-0063794 in comparison to PTEN-null cell lines, such as LNCaP.

### Treatment with AZD7328 and KU-0063794 reduces levels of phospho-biomarkers and induces cell cycle arrest in LNCaP cells

**Figure 2:**
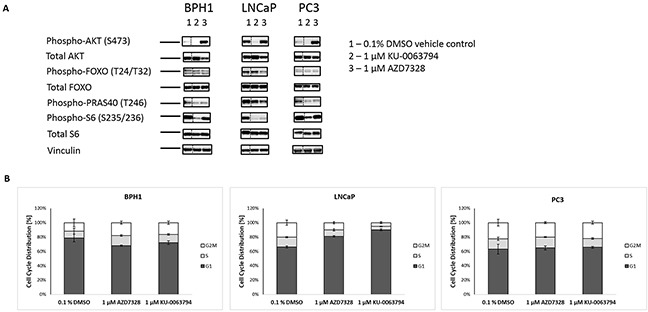
Inhibition of AKT and mTOR kinase reduces phospho-biomarker expression and induces cell cycle arrest in LNCaP cells **(A)** Prostate cell lines BPH1, LNCaP and PC3 were treated with either 1 γM AZD7328 or 1 γM KU-0063794 for 2 hours to measure acute response in change of biomarkers phosphorylation status. Following treatment, cells were lysed on ice, spun down and supernatant was collected. 20 γg of protein was loaded and run on 4-12% Bis-Tris polyacrylamide gel. Resolved protein was then electrotransferred to PVDF membranes and immunostained with antibodies against Phospho-AKT (S473), Phospho-FoxO1/FoxO3a (T24/T32), Phospho-PRAS40 (T246), Phospho-S6 (S235/236), total AKT, FoxO3a, total S6 and Vinculin, which was used as a loading control. Dashed line separating lane 1 (0.1% DMSO control) from the remaining 2 lanes indicates where images were cropped. **(B)** Cell cycle distribution was determined in BPH1, LNCAP and PC3 cells. Cells were treated with either 1 γM AZD7328 or 1 γM KU-0063794 for 72 hours. Following treatment, cells were washed with PBS, then harvested, fixed with 70% ice-cold ethanol, and stained with propidium iodide and analysed by flow cytometry. Control cells were treated with 0.1% DMSO. The signal was recorded in the PE channel, and debris were excluded from the analysis.

In order to validate the specificity of the inhibitors on the PI3K/AKT/mTOR pathway, we then determined the activity of selected phospho-biomarkers, by western blotting. PTEN-positive (BPH1) and PTEN-negative (LNCaP and PC3) cell lines were treated with 1 μM AZD7328 and KU-0063794 for 2 hours. The results showed a reduction in phospho-PRAS40 (T246), which is a substrate of AKT kinase, in all the cell lines, following treatment with AZD7328 (Figure 2A). However, phospho-FOXO (T24/T32) levels decreased only in LNCaP cells. Treatment with KU-0063794 resulted in a significant reduction of phospho-S6 (S235/236) (mTORC1 substrate) and phospho-AKT (S473) (mTORC2 substrate) in all three cell lines. This result confirmed the specificity of the inhibitors and their efficacy at a concentration of 1 μM, especially in LNCaP cells, where reduction of all respective biomarkers was observed.

Next, the effect of AKT or mTOR inhibition on cell cycle distribution was analysed. The results showed that the malignant LNCaP cells were the most affected. The majority of cells (86%) arrested in G0/G1 following 72-hour treatment with 1 μM KU-0063794, and 81% after treatment with 1 μM AZD7328, compared to 65% for the vehicle control (Figure 2B). Cell cycle distribution in BPH1 and PC3 cells did not change significantly after treatment with either of the inhibitors.

### Treatment of patient-derived cultures with AZD7328 induces autophagy and an increase in Phospho-ERK1/2 levels

**Figure 3:**
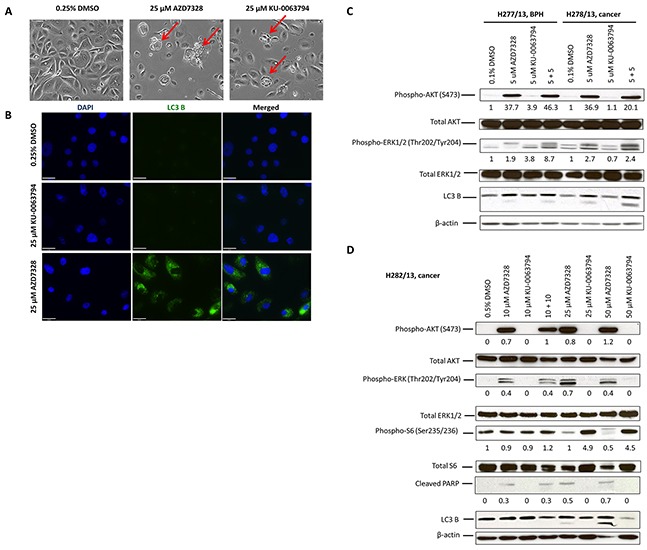
Autophagy is induced and phospho-ERK1/2 levels increase in patient-derived cultures after treatment with AKT inhibitor **(A)** Phase contrast images of primary cells (H282/13) from a Gleason 7 (4+3) prostate cancer, treated with 0.5% DMSO (left), 25 μM AZD7328 (middle) and 25 μM KU-0063794 (right) for 72 hours. Increased vacuolization of AZD7328-treated cells and membrane blebbing of KU-0063794-treated cells are indicated (red arrows). Control cells were treated with 0.5% DMSO. **(B)** LC3 B expression increases in primary prostate cancer cells H310/13 (GL7) following treatment with AZD7328. Expression of the autophagy marker, an LC3 B, was determined using Immunofluorescence. The cells were seeded into collagen I-coated 8-well chamber slides and treated with either 25 μM AZD7328 or 25 μM KU-0063794 for 96 hours and then fixed in 4% PFA. Control cells were treated with 0.25% DMSO. Scale bar on the phase contrast images represents 200μm and on the fluorescent images 63 μm. **(C)** Phospho-ERK1/2 levels increase in prostate primary cultures following treatment with AZD7328 and KU-0063794. Primary BPH (H277/13) and cancer cells (H278/13) were treated with 5 μM AZD7328, 5 μM KU-0063794 or 5 μM AZD7328 + 5 μM KU-0063794 for 72 hours. Westerns were carried out and biomarkers detected (phospho-AKT (S473), total AKT, phospho-ERK1/2 (Thr202/Tyr204), total ERK1/2 and LC3 B). Vehicle control cells were treated with 0.1% DMSO. Staining with β-actin antibody was used as a loading control. The bands were quantified using Image J software and the expression normalized to the vehicle control; the values are shown below the blot. **(D)** Primary prostate cancer cells H282/13 were treated for 72 hours with up to 50 μM of AZD7328 and KU-0063794 or a 10 μM AZD7328 + 10 μM KU-0063794 combination of both. Westerns were carried out and biomarkers detected (phospho-AKT, total AKT, phospho-ERK1/2, total ERK1/2, phospho-S6, total S6, cleaved PARP and LC3 B). Vehicle control cells were treated with 0.5% DMSO. Staining with β-actin antibody was used as a loading control. The bands were quantified using Image J software and the expression normalized to β-actin; the values are shown below the blot.

As there was more variability in primary cultures’ response to low concentrations of the inhibitors, we next investigated whether treatment with higher concentrations (10 – 50 μM) would result in cell death of the patient-derived cultures. A 72-hour treatment resulted in dramatic morphological changes, which were very specific for each inhibitor (Figure 3A). Treatment with 25 μM AZD7328 increased vacuolation of a cancer culture (H282/12), indicative of autophagy (Figure 3A, middle panel). In contrast, treatment of the same cells with 25 μM KU-0063794, caused membrane blebbing in a number of cells, indicative of apoptosis (Figure 3A, right panel). To follow up these observations, expression of an autophagy marker, LC3 B was analysed by immunofluorescence. The results showed evidence of autophagosomes in the cytoplasm in the cells treated with AZD7328, but not with KU-0063794 (Figure 3B). Autophagy was also measured by Western blot; conversion of LC3B-I to LC3B-II is suggestive of autophagy, which was observed after treatment with AZD7328 (Figure 3C and 3D). In addition, apoptosis was measured using cleaved PARP (Figure 3D). The results also demonstrated an increase in cleaved PARP levels in cells treated with AZD7328, but not KU-0063794 (Figure 3D), suggesting that despite activation of autophagy, cells died via apoptosis.

Additionally, treatment with AZD7328 caused an increase in phospho-ERK1/2 levels in all analysed cultures (Figure 3C and 3D), indicating an activation of the MAPK pathway in those cells. Treatment with KU-0063794 did not increase levels of phospho-ERK1/2 in any of the cancer cultures. However, an increase in MAPK pathway activity was observed in KU-0063794-treated BPH cultures (Figure 3C).

As EGF is an activating ligand of the MAPK pathway and is a component of primary cell culture media used in our laboratory, we sought to determine whether the EGF in the culture medium affected the outcome of treatment with the inhibitors. Selected phospho-biomarker levels were determined in H268/12 (BPH culture) following treatment with 5 μM AZD7328, 5 μM KU-0063794 or a combination of 5 + 5. The results showed that treatment with AZD7328 resulted in a 9-fold increase in phospho-AKT in the presence of EGF, whereas in the absence of EGF, a 12.1-fold increase in expression was observed (Supplementary Figure 3). Phosphorylation of ERK1/2 increased by 2.8-fold following treatment with AZD7328 (in the presence of EGF) and by 1.2 in the absence of EGF, which suggests that the ERK activation may be mediated via signalling from growth factors, such as EGF. However, phosphorylation of S6 remained at a comparable level following treatment, either in the presence or absence of EGF. We concluded that addition of EGF to the stem cell media affected, but did not mask the outcome of treatment. Therefore, in subsequent experiments EGF was included in the culture medium for primary cells.

### Combined treatment with AZD7328 and MEK1/2 inhibitors further enhances phosphorylation of ERK1/2 in patient-derived cultures but induces senescence

**Figure 4:**
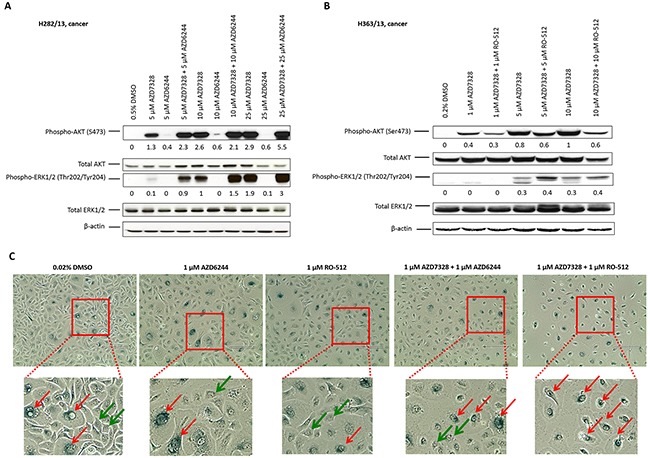
Senescence is induced following combination treatment with AZD7328 and MEK1/2 inhibitors Primary cancer cultures were treated with a range of concentrations of AZD6244, AZD7328 or combinations of AZD6244 + AZD7328 **(A)** or RO-512 + AZD7328 **(B)** for 72 hours. Westerns were carried out and biomarkers detected (phospho-AKT, total AKT, phospho-ERK1/2 and total ERK1/2). Vehicle control cells were treated with 0.5% DMSO **(A)** or 0.2 % DMSO **(B).** The bands were quantified using Image J software and the expression normalized to β-actin; the values are shown below the blot. **(C)** Primary BPH culture H373/13 was treated with either 1 μM AZD6244, 1 μM RO-512 or a combination of 1 μM AZD6244 + 1 μM AZD7328 or 1 μM RO-512 + 1 μM AZD7328 for 72 hours. Following treatment, cells were fixed and stained according to the manufacturer's instructions and phase contrast images were taken. β-galactosidase – positive cells were observed (red arrows) as well as β-galactosidase negative cells (green arrows). Vehicle control cells were treated with 0.02% DMSO. Scale bar represents 400 μm.

To establish whether inhibition of MEK1/2 would be sufficient to overcome the compensatory effect of MAPK pathway activation, following inhibition of AZD7328, two small molecule inhibitors of MEK1/2, AZD6244 and RO-5126766, were tested. Following confirmation of inhibitory activity of both MEK1/2 inhibitors in prostate epithelial cultures (Supplementary Figure 4), combination treatment with AZD7328 + AZD6244 or AZD7328 + RO-512 were performed in patient-derived cancer cultures, for 72 hours. The results from Western blotting showed a further increase in phospho-ERK1/2 levels after treatment with either of the combinations (Figure 4A and 4B), even after treatment with a high concentration (up to 25 γM) of the MEK1/2 inhibitors. This suggests that despite confirmed inhibitory activity against MEK1/2 kinase, neither AZD6244 nor RO-512 inhibitor is capable of preventing ERK1/2 phosphorylation following compensatory activation of MAPK pathway. However, despite this increase in phospho-ERK1/2 levels, the effect of combined treatment results in cellular senescence as detected by the acidic β-galactosidase staining (Figure 4C).

### Combined inhibition of AKT and mTOR decreases tumour frequency in a patient-derived xenograft model

To determine whether AKT and mTOR blockade could inhibit tumour growth, tumour cells were harvested from a human prostate cancer xenograft, (derived from a patient with hormone-naïve Gleason 7 disease), and were subsequently treated with either 10 μM AZD7328, 10 μM KU-0063794 or a combination of 10 + 10, for 72 hours. Following treatment, the cells were engrafted (at limiting dilutions) into Rag2^−/−^γC^−/−^mice to determine the effect of treatment on tumour frequency. The results showed that xenograft tumour cells were still able to initiate tumours following treatment with either inhibitor or a combination of both (Table 4). However, tumour frequency was significantly reduced with the combination treatment (*P* =0.010) (Table 5). In contrast, neither AZD7328 nor KU-0063794 blockade affected tumour outgrowth.

**Table 1:** EC_50_ for AZD7328 and KU-0063794 in prostate cell lines

Cell line	Origin	PTEN status	EC_50_ [μM]	
			AKT inhibitor	mTOR inhibitor
**BPH-1**	Benign prostate	+	>50	5.8 (3.4-8.3)
**P4E6**	Localised prostate tumour	+	>50	10.2 (7.2-13.2)
**LNCaP**	Lymph node metastasis	-	0.4 (0.3-0.5)	1.03 (0.78-1.28)
**PC3**	Bone metastasis	-	11.0 (6.2-15.8)	0.6 (0.3-0.9)

**Table 4:** Tumour initiation frequency is lower in xenograft cells treated with a combination of AKT and mTOR inhibitor

Number of inoculated cells	10 000 cells	1000 cells	100 cells	10 cells	1 cell	Tumour initiation frequency(95% CI)
Treatment						
**0.2% DMSO**	2/2	2/2	2/2	1/2	0/2	16.6 (98.9-3.16)
**10 γM AZD7328**	2/2	2/2	2/2	0/2	0/2	43.8 (201.1-9.84)
**10 γM KU-0063794**	2/2	2/2	2/2	1/2	0/2	16.6 (98.9-3.16)
**10 +10 combination**	2/2	2/2	0/2	0/2	0/2	434.6 (2008.4-94.36)

**Table 5:** Pairwise tests for differences in tumour frequencies

Group 1	Group 2	p value
**AZD7328**	Combination	0.071
**AZD7328**	DMSO	0.443
**AZD7328**	KU-0063794	0.443
**Combination**	DMSO	0.010
**Combination**	KU-0063794	0.010
**DMSO**	KU-0063794	1

An additional effect of treatment with a combination of the inhibitors was an 8-day increase in the tumour latency compared to vehicle control (38 days and 30 days, respectively). There was no difference in tumour latency following treatment with either inhibitor alone.

## DISCUSSION

The PI3K/AKT/mTOR and the Androgen Receptor signalling pathways are important drivers of prostate cancer growth and progression. Despite progress in prostate cancer treatment with the development of second generation anti-androgen agents, patient outcome remains unsatisfactory. Following the development of endocrine resistance, patients are typically treated with docetaxel, which is effective in 40 – 55 % of patients [4, 35–37]. Targeting of the PI3K/AKT/mTOR pathway has been investigated in a number of studies as an alternative treatment for patients with over-activation of the pathway [38, 39]. Since a reciprocal feedback between the AR and the PI3K pathways has been demonstrated in prostate cancer, a combination of inhibitors targeting the PI3K pathway and anti-androgens, such as abiraterone and enzalutamide is currently in clinical trials [8, 40]. However, targeting androgen-dependent prostate cells does not eradicate the stem cell component of prostate tumours, which we and others have shown to have an AR-negative phenotype [24, 28, 41]. Therefore alternative combinations of drugs are urgently required if we are to progress in the treatment of prostate cancer and the potential of PI3K pathway inhibitors has to be fully elucidated.

The aim of this study was to determine the importance of the PI3K/AKT/mTOR pathway in human prostate cancer. This was performed by targeting the pathway with AKT and mTOR inhibitors in prostate cancer cell lines, primary cell cultures and a ‘near-patient’ xenograft. Activity of the small molecule inhibitors against AKT and mTOR kinases used in this study have also been tested in a number of other tumour types [42, 43].

The results of treatment with AZD7328 and KU-0063794 showed that PTEN-negative LNCaP cells were the most susceptible to treatment. Treatment of primary cultures (derived from patients with BPH and cancer) confirmed variability in response between patient samples, even from patients with the same Gleason grade. In contrast to established prostate cancer cell lines, PTEN protein expression was observed in all primary cells investigated, including Gleason 8 tumours. Although there was variability in the levels of PTEN expression, none of the cultures showed a complete loss of PTEN. However, 3 out of 7 (43%) patient-derived cultures had mutations in exon 5 of the *PTEN* gene and 2 out of 7 (28.6%) showed loss of heterozygosity in tumour DNA, in comparison to matched lymphocyte DNA. PTEN loss is typically observed in 70-80% of castration resistant prostate tumours [32, 33, 44]. This discrepancy can be explained because the majority of cultures used in this study were derived from treatment naïve patients with early stage disease, whereas PTEN is more frequently lost in advanced prostate cancers. Only 12% of radical prostatectomy samples show complete loss of PTEN [45]. Nevertheless these cultures are relevant because it is imperative that combinations of inhibitors that delay progression to advanced disease, after initial androgen deprivation therapy or radiotherapy, are identified.

Targeting the PI3K/AKT/mTOR pathway has been proposed as a clinical strategy to eliminate cancer stem cells. For example, treatment of anaplastic thyroid cancer with a PI3K inhibitor, LY294002, attenuated the self-renewal of cancer stem cells and induced cell differentiation [46]. Similar findings were also reported in acute myeloid leukaemia and in breast cancer stem cells, where it was shown that the PI3K/AKT/mTOR pathway was critical for cancer stem cell proliferation and survival [47, 48]. Moreover, treatment with a chemotherapeutic drug cisplatin, results in a significant increase in breast cancer stem cells, which could be partially attributed to the activation of the PI3K/AKT/mTOR pathway [49]. However, combined treatment with cisplatin and the mTOR inhibitor rapamycin synergistically inhibited cancer stem cell-mediated primary and metastatic cancer growth [49]. These recent findings emphasise the need to test combination treatments, as a more successful approach to targeting cancer stem cells. Our findings suggest that despite differences between patients’ responses, targeting stem cells with a combination of AZD7328 and KU-0063794 might potentially be more successful than a monotherapy with either drug alone. Further investigation is needed to identify differences between treatment responders and non-responders, in a more efficient stratification of patients in the clinic.

Treatment with high concentrations (≥ 25 μM) of AKT and mTOR inhibitors induced dramatic morphological changes, such as vacuolation of cells and membrane blebbing. Although these concentrations are much higher than exposures achieved in man by AKT and mTOR inhibitors of similar potency, the biology invoked as a consequence of greater target inhibition is of interest. Membrane blebs are spherical outgrowths of the plasma membrane that occur upon membrane detachment from the underlying cytoskeleton and are indicative of apoptosis [50]. However, we observed no increase in cleaved PARP expression after mTOR inhibitor treatment. This could be due to a minority of cells showing apoptotic features, and therefore being undetectable by western blotting. Increased vacuolation of cells following treatment with the AKT inhibitor was morphologically indicative of autophagy and the formation of autophagosomes. Autophagy is a cellular catabolic degradation process in response to starvation or stress, in which cellular organelles, proteins and cytoplasm are digested and recycled to maintain metabolism [51, 52]. A clear increase in the conversion of LC3-B I to LC3-B II (autophagy marker) demonstrated by both western blotting and immunofluorescence confirmed the morphological evidence. Treatment with the ATP-competitive AKT inhibitor (AZD7328) does induce autophagy in human bladder cancer cell lines, which can be overcome by treating the cells with chloroquine [53]. However, autophagy can also prevent tumourigenesis, acting as a tumour suppressor [52, 54]. Accumulation of the autophagy substrate p62/SQSTM1 is sufficient to both activate the MAPK pathway and increase cell proliferation in PI3K-expressing mammary 3D structures [54]. Increased cell proliferation and MAPK activation, due to inhibition of autophagy, was only evident under 3D culture conditions, and not in monolayer cells [54]. In 3D, the cells treated with AZD7328 showed the expected increase in autophagy, as well as activation of the MAPK pathway.

We observed activation of the MAPK pathway following treatment with AZD7328, and so the primary cultures were also treated with a combination of AZD7328 and MEK1/2 inhibitors to determine the outcome of inhibiting this compensatory pathway. Surprisingly, a combined treatment with AZD7328 and either AZD6244 or RO-512 resulted in an increased phosphorylation of ERK1/2 to even greater levels than treatment with AZD7328 alone. Davies and colleagues have previously demonstrated activity and efficiency of the AZD6244 in a large panel of human cancer cell lines as well as in xenografts derived from them [55]. It has been reported that a majority of cell lines with a mutation in *KRAS*, *NRAS* or *BRAF* genes showed a significantly increased sensitivity to MEK inhibition, resulting in cell growth inhibition and induction of apoptosis [55]. In the human colon carcinoma xenograft HCT-116, a combination treatment of the AZD6244 and docetaxel resulted in enhanced tumour growth inhibition, in comparison to monotherapy [56]. However, the rate of mutations in Ras/MEK/ERK pathway in clinical prostate cancer is very low; only up to 7% of clinical samples have activating point mutations of Ras and around 4 % are positive for BRAF, the most common activating mutation of RAF [57]. Similarly, efficacy of RO-512 has been demonstrated in a number of human cancer cell lines and also in the HCT-116 xenograft model [58]. However, in our patient-derived cultures, neither of the MEK1/2 inhibitors was able to overcome the compensatory ERK1/2 activation caused by inhibition of AKT.

It is unclear why combination treatment of prostate cancer primary cells with AZD7328 and MEK1/2 inhibitors did not reduce ERK1/2 phosphorylation. A possible explanation is that we could be observing a profound stress response where other MAPKs are activated and although phospho-ERK1/2 antibody we used does not cross-react with the corresponding phosphorylated residues of either JNK/SAPK or p38 MAP kinases, this phenomenon will have to be investigated further. However, acidic β-galactosidase staining of treated cultures revealed that the cells became senescent after the combination treatment. Senescence is frequently triggered in tumours in response to chemotherapy and radiotherapy treatment in cancer patients [59, 60] and it has been demonstrated that PTEN-loss-induced cellular senescence inhibits tumorigenesis *in vivo* in a human xenograft model of prostate cancer [61]. Induction of senescence in cancer cells could indeed be a good therapeutic strategy for treatment of prostate cancer if it established stable disease [61].

The PI3K/AKT/mTOR and the Ras/MEK/ERK pathways are the two major hyper-activated pathways, which promote cell proliferation, survival and metastasis in human cancer [11, 62]. Significant activation of MAPK signalling has been observed in both primary and in metastatic cancer [63]. Activation of the MAPK pathway has been implicated in resistance to the PI3K/AKT/mTOR signalling inhibition in other studies [64]. Here, we present for the first time, evidence that the MAPK pathway acts as a compensatory pathway in primary prostate epithelial cell cultures following AKT inhibition. However, the possibility of a third compensatory pathway cannot be discounted by our data.

Despite our observation that the Ras/MEK/ERK compensatory pathway is activated in primary cells following the PI3K/AKT/mTOR inhibition, the *ex vivo* treatment of the PDX with a combination of KU-0063794 (mTOR inhibitor) and AZD7328 (AKT inhibitor) showed an effective reduction in tumour outgrowth. Therefore this combination treatment alongside anti-androgen therapy has the potential to be more effective than single treatments. However, due to the compensatory pathway and the further possibility of as yet unknown effects, the outcome of drug combinations may not be predictable and may have to be determined on a patient specific basis. The utilisation of primary cell cultures from multiple individual patients will allow elucidation of mechanisms of action of drugs, mechanisms of resistance and variation in patient response, at a level of which cannot be predicted using the standard panel of available prostate cancer cell lines.

## MATERIALS AND METHODS

### Inhibitors

The AKT inhibitor AZ7328, mTOR inhibitor KU-0063794 and MEK inhibitor Selumetinib (AZD6244; ARRY-142886) were obtained from AstraZeneca, and the MEK1/2 inhibitor RO-5126766 (RO-512) was purchased from MedKoo Biosciences, Inc., NC, USA. AZ7328 is an ATP-competitive, selective inhibitor of AKT [53] that inhibits all three AKT isoforms with an IC50 of < 50 nM in isolated enzyme assays, and inhibits the phosphorylation of GSK3β in PTEN null MDA-MB-468 breast cancer cells with an IC50 of 91 nM. KU-0063794 is a highly selective inhibitor of mTOR kinase [43] and Selumetinib is a selective, non-ATP competitive inhibitor of MEK1/2 kinases [55]. RO-5126766 is an allosteric MEK inhibitor which has been shown to additionally supress feedback induction of RAF-dependent MEK phosphorylation [58].

### Culture of cell lines

Human prostate cell lines used in the experiments included: BPH1, P4E6, LNCaP and PC3 cells. LNCaP and PC3 cells were purchased from American Type Culture Collection (ATCC), BPH1 were a gift from Dr Hayward (NorthShore University HealthSystem Research Institute), and P4E6 cells were derived in our laboratory [65]. BPH1 and LNCaP cells were cultured in Roswell Park Memorial Institute-1640 (RPMI) (Invitrogen Ltd) supplemented with 5% or 10% Foetal Calf Serum (FCS) (PAA), respectively, and 2 mM L-Glutamine (Invitrogen Ltd). PC3 cells were cultured in Ham's F-12 medium (Lonza) supplemented with 7% FCS and 2 mM L-Glutamine. P4E6 cells were cultured in Keratinocyte Serum-Free Medium (KSFM) (Invitrogen Ltd) supplemented with 2% FCS, 2 mM L-Glutamine, 5 ng/ml Epidermal Growth Factor (EGF), 50 μg/ml bovine pituitary extract (Invitrogen Ltd). All cell lines were cultured in T25 or T75 cell culture flasks (Corning) at 37°C in 5% CO_2_.

### Tissue collection and culture of tumour cells

Prostate tissue was obtained from patients undergoing radical prostatectomy or transurethral resection of the prostate (TURP) (Table 3), with informed patient consent and approval from the local Research Ethics Committee (07/H1304/121). All patient samples were anonymized. Epithelial cultures were prepared as described previously [31]. Cell cultures were maintained in stem cell media (SCM), which consisted of keratinocyte growth medium supplemented with 5 ng/ml Epidermal Growth Factor (EGF), 50 μg/ml bovine pituitary extract (Invitrogen Ltd), 2 ng/ml stem cell factor (First Link), 100 ng/ml cholera toxin (Sigma-Aldrich Company Ltd) and 1 ng/ml GM-SCF (First Link). Epithelial cells were cultured on collagen I – coated plates in the presence of irradiated (60 Gy) STO (mouse embryonic fibroblasts) cells.

**Table 3:** Patient data summary

Patient ID	Operation	Diagnosis (Gleason grade)	Age	Hormone therapy
**H233/12**	Radical prostatectomy	Normal	61	Naive
**H268/12**	TURP	BPH	62	-
**H277/13**	TURP	BPH	68	-
**H313/13**	TURP	BPH	65	-
**H278/13**	Radical Prostatectomy	Cancer - Gleason 6 (3 + 3)	72	Naive
**H315/13**	Radical Prostatectomy	Cancer - Gleason 6 (3 + 3)	62	Naive
**H237/12**	Radical Prostatectomy	Cancer - Gleason 7 (3 + 4)	66	Naive
**H240/12 RA+RB**	Radical Prostatectomy	Cancer - Gleason 7 (3 + 4)	66	Naive
**H252/12**	Radical Prostatectomy	Cancer - Gleason 7 (3 + 4)	60	Naive
**H310/13 R+L**	Radical Prostatectomy	Cancer - Gleason 7 (3 + 4)	67	Naive
**H329/13 LB**	Radical Prostatectomy	Cancer - Gleason 7 (3 + 4)	53	Naive
**H373/13**	Radical Prostatectomy	Cancer - Gleason 7 (3 + 4)	82	Naive
**H377/13**	Radical Prostatectomy	Cancer - Gleason 7 (3 + 4)	60	Naive
**H233/12**	Radical Prostatectomy	Cancer - Gleason 7 (4 + 3)	61	Naive
**H282/13 RA+RB**	Radical Prostatectomy	Cancer - Gleason 7 (4 + 3)	62	Naive
**H236/12**	Radical Prostatectomy	Cancer - Gleason 8 (4 +4)	61	Naive
**H163/12**	Channel TURP	Cancer - Gleason 8 (4 +4)	65	Yes
**H239/12 RB**	Radical Prostatectomy	Cancer - Gleason 9 (4 + 5)	50	Naive
**H149/12**	Channel TURP	Cancer - Gleason 9 (4 + 5)	78	Yes
**H224/12**	Channel TURP	Cancer - Gleason 9 (4 + 5)	76	Yes
**H271/12**	Channel TURP	Cancer - Gleason 9 (4 + 5)	86	Yes
**H135/11**	Channel TURP	Cancer - Gleason 9 (5 + 4)	56	Yes

### Generation of xenografts and isolation of tumour cells for *ex-vivo* treatment

All animal work was approved by the University of York Animal Procedures and Ethics Committee and performed under a United Kingdom Home Office License. Human prostate cancer tissue was obtained from a patient with hormone naïve, Gleason 7 disease. Tumour Xenografts were derived according to the method outlined in Kroon et al., 2013 [66]. Xenografts were maintained subcutaneously in BALB/c/Rag2^−/−^γC^−/−^ mice, which are highly receptive for engrafting of human tissue due to deficiencies in T, B and NK cell development [67].

15 mm^3^ tumours were harvested from humanely euthanized mice and human Lin-/CD31-, were isolated according to Kroon et al., 2013 [66]. Tumour cells were resuspended in D10 medium, plated onto collagen I – dishes and treated for up to 72 hours with AKT and mTOR inhibitors. Following treatment, the cells were resuspended in Matrigel and injected subcutaneously into the flanks of a Rag2^−/−^γC^−/−^, 2×10^5^ irradiated STO were used as carrier cells. Mice were monitored weekly for tumour growth. Once the tumours were detectable they were measured every 2-3 days using a digital calliper (Duratool DC150). Tumour volume (mm^3^) was calculated using the ellipsoidal formula: (length x width^2^)/2. Tumour initiation frequency was calculated using Extreme Limiting Dilution Analysis software available online at (http://bioinf.wehi.edu.au/software/elda/), which provided an estimate of frequency with 95% confidence intervals and includes the Pearson's chi-square test for pairwise analysis to determine differences between treatment groups.

### Treatment of subpopulations of primary cell cultures

Primary cells were seeded onto collagen I-coated 10 cm dishes, 24 hours prior to treatment. Cells were treated with increasing concentrations (0.1 μM to 50 μM) of AZD7328, KU-0063794 or MEK1/2 inhibitors either alone or in combination for up to 72 hours. Control cells were treated with DMSO. Following treatment, α_2_β_1_^hi^/CD133^+^(stem-like cells), α_2_β_1_^hi^/CD133^−^ (transit amplifying, TA) and α_2_β_1_^low^ (committed basal, CB) cells were isolated by MACS (Miltenyi Biotec Ltd.), as described previously [68, 69]. Briefly, cell subpopulations were enriched and selected from primary cell cultures, first using collagen adherence to separate α_2_β_1_^hi^ and α_2_β_1_^low^ cells. This was followed by incubating the α_2_β_1_^hi^ cells with anti-human CD133 microbeads and selecting CD133+ cells using MACS magnetic bead selection (Miltenyi Biotec Ltd). This results in three cell subpopulations – α_2_β_1_^hi^/CD133^+^(stem-like cells), α_2_β_1_^hi^/CD133^−^ (transit amplifying, TA) and α_2_β_1_^low^ (committed basal, CB) cells.

### PTEN mutational analysis and loss of heterozygosity (LOH) analysis

The Polymerase chain reaction (PCR) was used to amplify matched lymphocyte and tumour DNA from the patient primary cultures to determine mutational status and loss of heterozygosity of the *PTEN* gene. Standard PCR conditions were adapted for the analysis of *PTEN* exon 5 [34]. PCR products were column purified using Qiagen Quick PCR clean up kit (Qiagen, UK) and then directly sequenced using *PTEN* exon 5 sequencing primers (Table 2). The presence of mutations was determined by using EditSeq software (DNASTAR).

Loss of heterozygosity in *PTEN* was assessed using two LOH probes described in Dahia et al [34]; they included a dinucleotide repeat in *PTEN* intron 2 (AFMa086wg9) (probe 1) and a G/T sequence polymorphism in *PTEN* intron 8 (probe 2). The reverse primer for intron 2 was labelled with a JOE fluorescent dye enabling PCR fragment analysis, which was performed using Applied Biosystems ABI3130XL and data analysed using Gene Mapper software (Life Technologies). LOH was identified in the heterozygous tumour samples as a reduction in one of the peaks, which corresponded to one of the alleles of the *PTEN* gene. The G/T polymorphism was analysed using digestion with restriction enzyme HincII (New England Biosciences), where the polymorphism is differentially cleaved in heterozygous and homozygous individuals.

### Agarose gel electrophoresis

PCR products were visualised using 1.5% agarose gel electrophoresis prepared with Tris-Acetate-EDTA buffer and run at 80V. The products of the HincII digestion reaction were separated in a 3% agarose gel in Tris-Borate-EDTA buffer and run at 100V.

### SDS-PAGE and Western blotting

Adherent and non-adherent cells were harvested and resuspended in Cytobuster protein extraction reagent (Novagen) supplemented with the addition of protease inhibitor cocktail (Roche) and phosphatase inhibitor cocktail (Roche). Whole cell protein lysates were quantified using the Bicinchoninic acid assay (BCA) protein assay kit (Thermo Scientific), according to manufacturer's instructions.

20 μg of total cell lysate was heated to 100°C for 10 min in sample buffer (4 x SDS loading buffer (10% (v/v) glycerol, 62.5 mM Tris-HCl pH 6.8, 1% (w/v) SDS, 65 mM DTT and bromophenol blue), and subjected to PAGE and electrotransfer onto Immobilon-P membrane (Millipore). Membranes were blocked for 1 hr with 10% blocking reagent (Roche) in TBS-Tween, followed by incubation with primary antibodies at 4°C for 2 hours or overnight. Antibodies were purchased from Cell Signaling Technology, unless otherwise specified, and included: AKT (pan) (#4691), phospho-AKT (Thr308) (#2965), phospho-AKT (Ser473) (#4060), 4E-BP1 (#9452), phospho-4E-BP1 (Thr37/46) (#2855), p44/42 MAPK (ERK1/2) (#4695), phospho-p44/42 MAPK (ERK1/2) (Thr202/Tyr204) (#4370), LC3-B (abcam, ab51520), PRAS40 (#2691), phospho-PRAS40 (Thr246) (#2997), PTEN (#9188), S6 Ribosomal Protein (2217), phospho-S6 (Ser235/236) (#2211), FoxO3a (#9467), phospho-FoxO1 (Thr24)/FoxO3a (Thr32) (#9464), Vinculin (Sigma V9131), β-actin (Sigma, A4700). Chemiluminescent detection was performed using the BM Chemiluminescence Blotting Substrate (POD) system (Roche). Labelled proteins were visualised using ECL Hyperfilm (Amersham) and manually processed using Kodak GBX developer and fixer solutions (SLS).

### Flow cytometry and cell cycle analysis

Cell lines and primary prostate cells were used for cell cycle analysis following treatment. Cells were fixed in ice-cold 70% Ethanol, then incubated on ice for 30 minutes and resuspended in PBS. RNase A (at 1 mg/ml final concentration) and propidium iodide (PI; 400 γg/ml final concentration) were added, and cells incubated at 37°C for 30 min. Cells were then placed on ice, analysed on a CyAn ADP flow cytometer (Dako Cytomation) and PI fluorescence was recorded in the PE channel. To prevent bleaching of the fluorescence, samples were protected from light where possible during the procedure. The results were analysed using the Summit v4.3 software.

### Immunofluorescence

Primary prostate cells were seeded at a density of 7.5 × 10^4^ per well in BD-Biocoat collagen I-coated 8-well chamber slides and incubated at 37°C at 5% CO_2_ for 24 hours before treatment. Following treatment, cells were fixed, permeabilised and stained as described previously [69]. Antibodies used were LC3-B (abcam, ab51520 at 1:2000) and secondary anti-rabbit Alexa Fluor 488 (Invitrogen, A-11034 at 1:300). Slides were mounted in Vectashield containing DAPI (Vector laboratories). Images were captured using a Nikon Eclipse TE300 fluorescent microscope (Nikon). Secondary only antibodies, where the primary antibody was replaced with PBS, were used as controls.

### β-Galactosidase staining assay

Cells were seeded onto 10 cm dishes and, following treatment, an acidic β-Galactosidase assay (Cell Signaling Technology) was performed as an indicator of senescence. Briefly, cells were washed once with PBS and fixed for 15 minutes at room temperature, washed twice with PBS, and then incubated with β-Galactosidase staining solution, at pH 6.1, overnight at 37°C in a dry incubator. The staining results were analysed using an Evos XL transmitted light microscope (AMG) at 10x and 20x magnification.

## SUPPLEMENTARY MATERIALS FIGURES


