# Supplementary material for: Inhibition of the PI3K/AKT/mTOR pathway activates autophagy and compensatory Ras/Raf/MEK/ERK signalling in prostate cancer
Source: Oncotarget. 2017 May 23;8(34):56698–713. doi: 10.18632/oncotarget.18082 (PMC5593594; doi:10.18632/oncotarget.18082)
Supplement: Supplementary file 1 [file oncotarget-08-56698-s001.pdf]

Inhibition of the PI3K/AKT/mTOR pathway activates autophagy and compensatory Ras/Raf/MEK/ERK signalling in prostate cancer

SUPPLEMENTARY MATERIALS

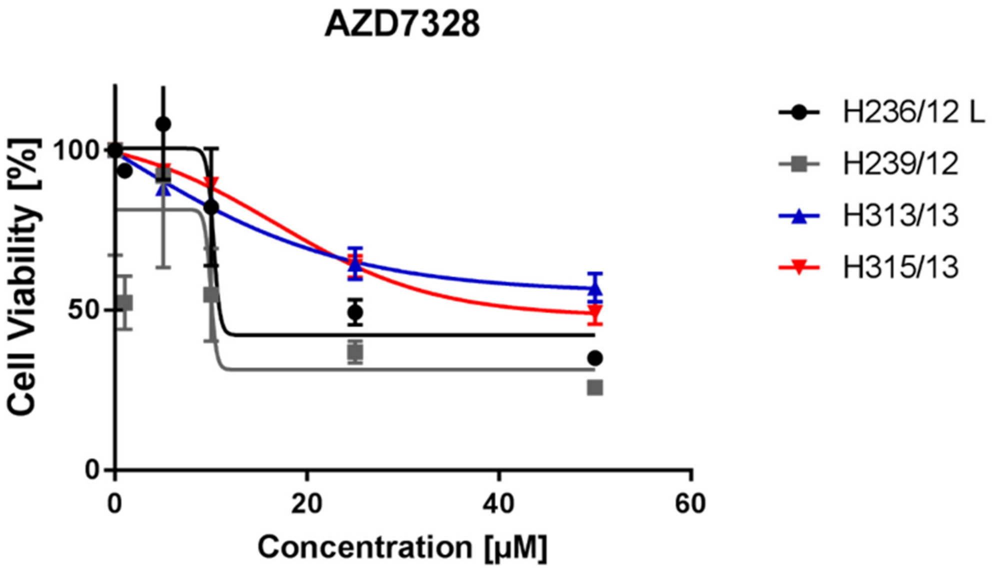

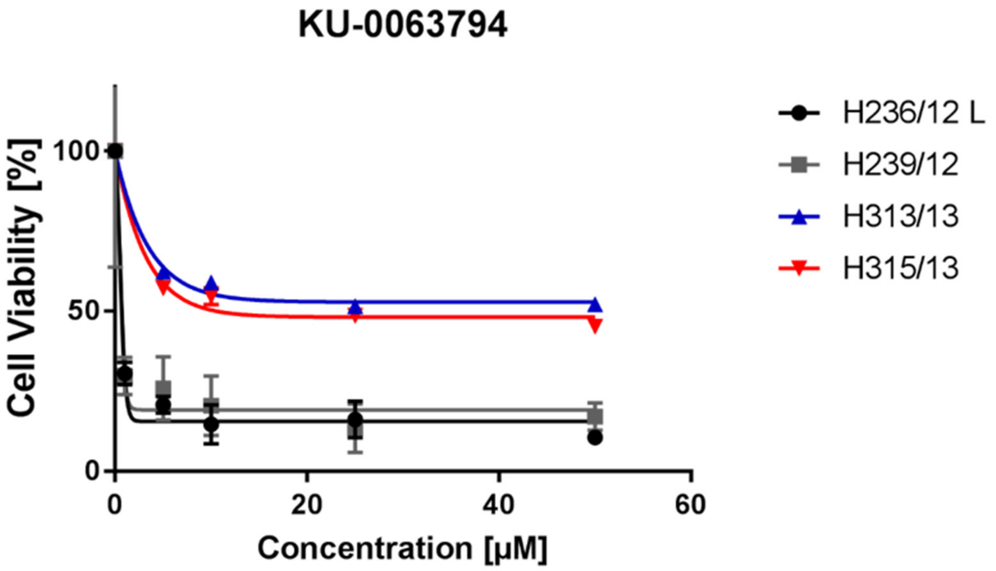

Supplementary Figure 1: Cell viability is reduced following treatment with AZD7328 and KU-0063794 in primary prostate cells. An MTS assay was performed with primary prostate cultures (derived from cancers, n=3 and from BPH, n=1) treated with increasing concentrations of AKT and mTOR inhibitors, for 72 hours. Cell viability was determined relative to the vehicle control (0.5% DMSO). The analysis was performed using GraphPad Prism 7.0 software. Error bars represent standard deviation of technical replicates.

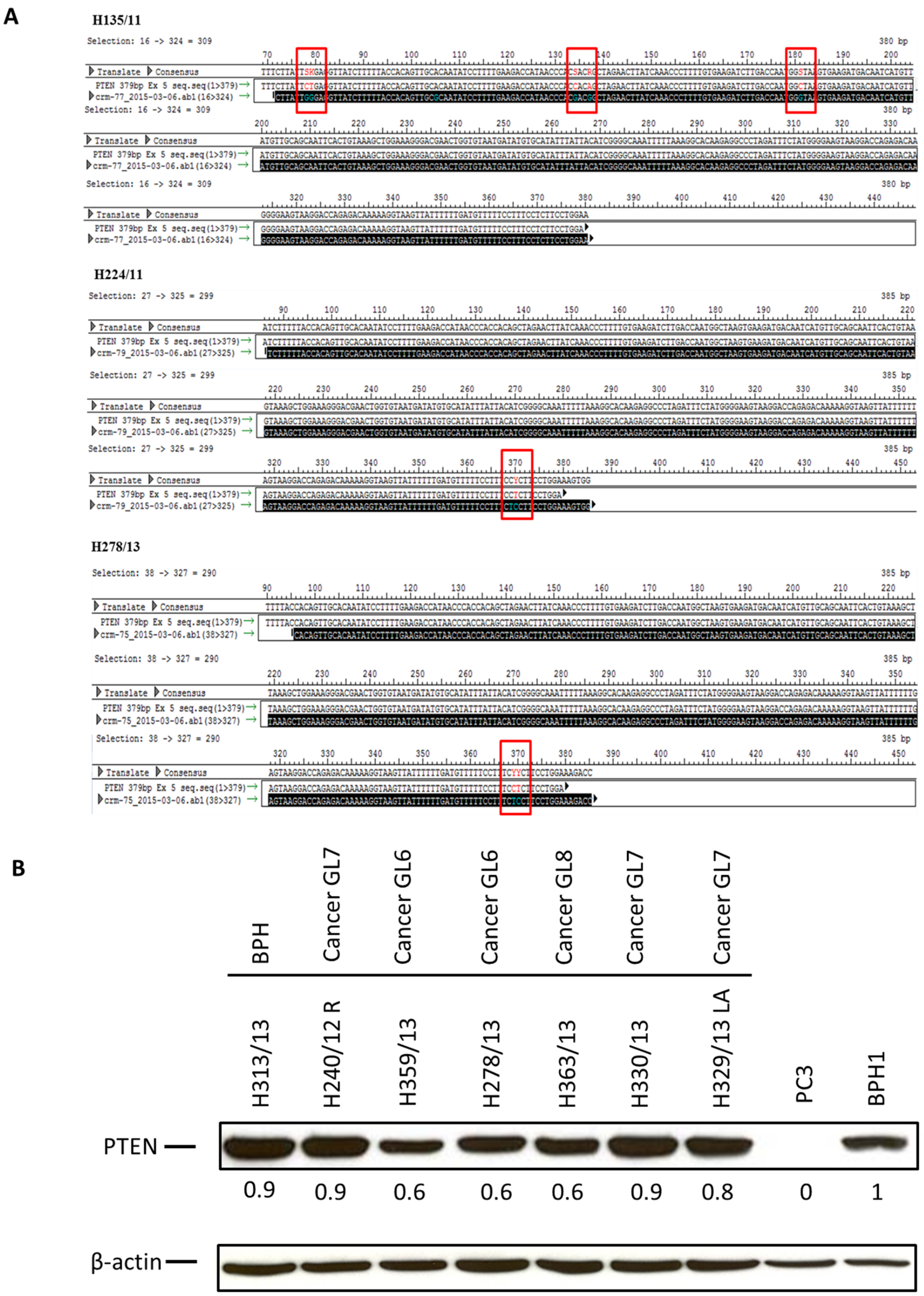

Supplementary Figure 2: PTEN is expressed in patient-derived epithelial cultures. (A) PTEN exon 5 sequencing analysis showed presence of a number of point mutations (highlighted in red boxes), resulting in missense mutations of the region encoding the phosphatase domain of the PTEN. The Polymerase chain reaction (PCR) was used to amplify matched lymphocyte and tumour DNA from the patient primary cultures to determine mutational status of the *PTEN* gene. Standard PCR conditions were adapted for the analysis of *PTEN* exon 5; PCR products were column purified using Qiagen Quick PCR clean up kit and then directly sequenced using *PTEN* exon 5 sequencing primers (Table 2). The presence of mutations was determined by using EditSeq software (DNASTAR). **(B)** PTEN protein expression was determined in primary cultures using western blotting. Whole cell lysates were prepared and 20 µg of protein was loaded per lane onto a 10% SDS gel, electrotransferred onto PVDF membranes and immuno-stained for PTEN protein. Whole lysates from BPH1 (PTEN-positive cell line) and PC3 (PTEN-negative cell line) were used as controls. Staining with β-actin antibody was used as a loading control. The bands were quantified using Image J software and the expression normalized to BPH-1 (positive control); the values are shown below the blot.

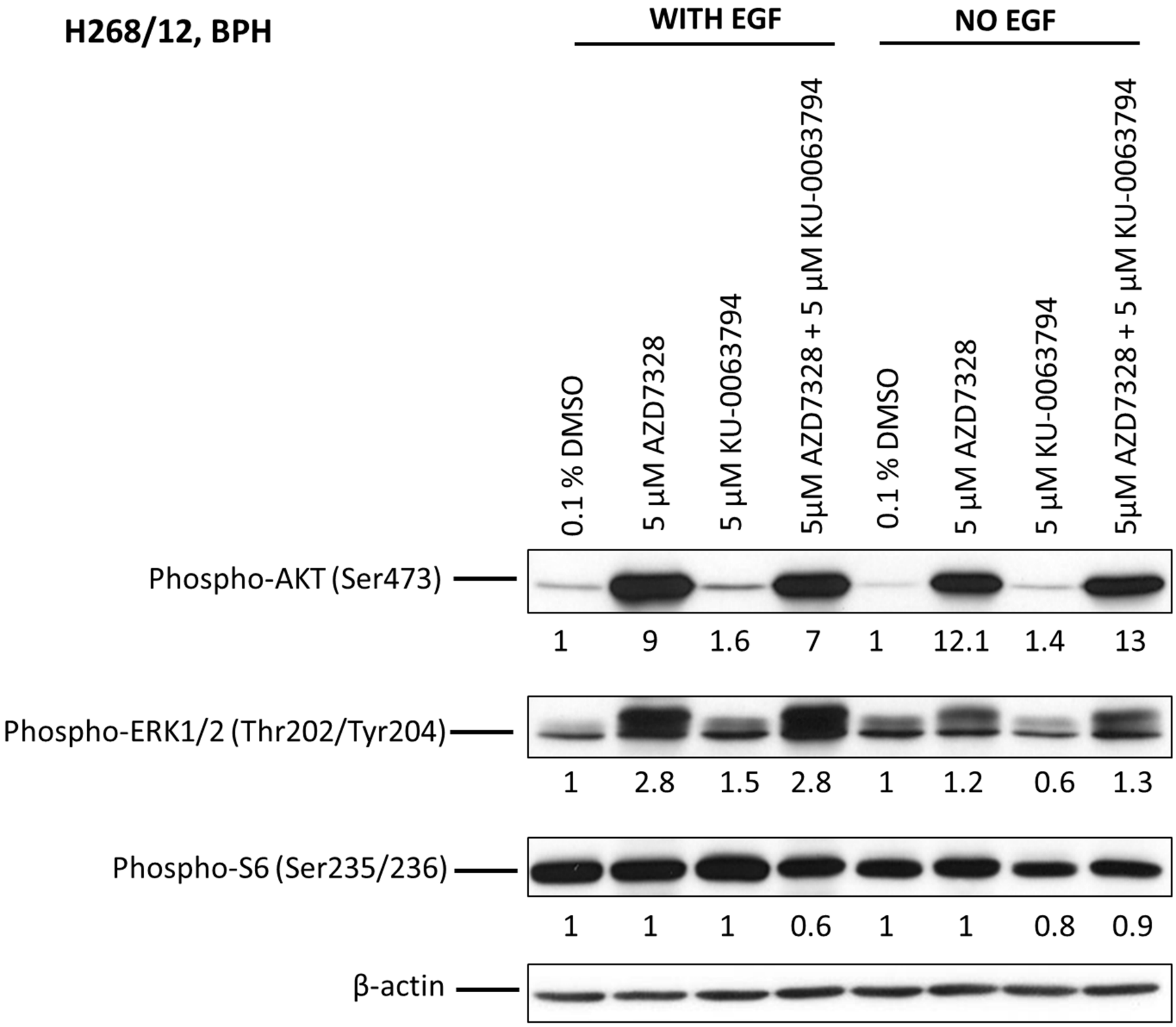

Supplementary Figure 3: Phospho-biomarker expression analysis following treatment with AZD7328 and KU-0063794 in presence or absence of EGF in culture media. A BPH (H268/12) culture was treated with either 5 µM of AZD7328 or 5 µM KU-0063794 alone or in a combination of 5 + 5 for 72 hours. Cells were subsequently collected, whole cell lysates prepared and quantified. 20 µg of protein was loaded per lane, electrotransferred to PVDF membrane and stained with antibodies against phospho-AKT (Ser473), phospho-ERK1/2 (T202/Tyr204) and phospho-S6 (Ser235/236). Staining with β-actin antibody was used as a loading control. The bands were quantified using Image J software and the expression levels normalized to the vehicle control; the values are shown below the blot.

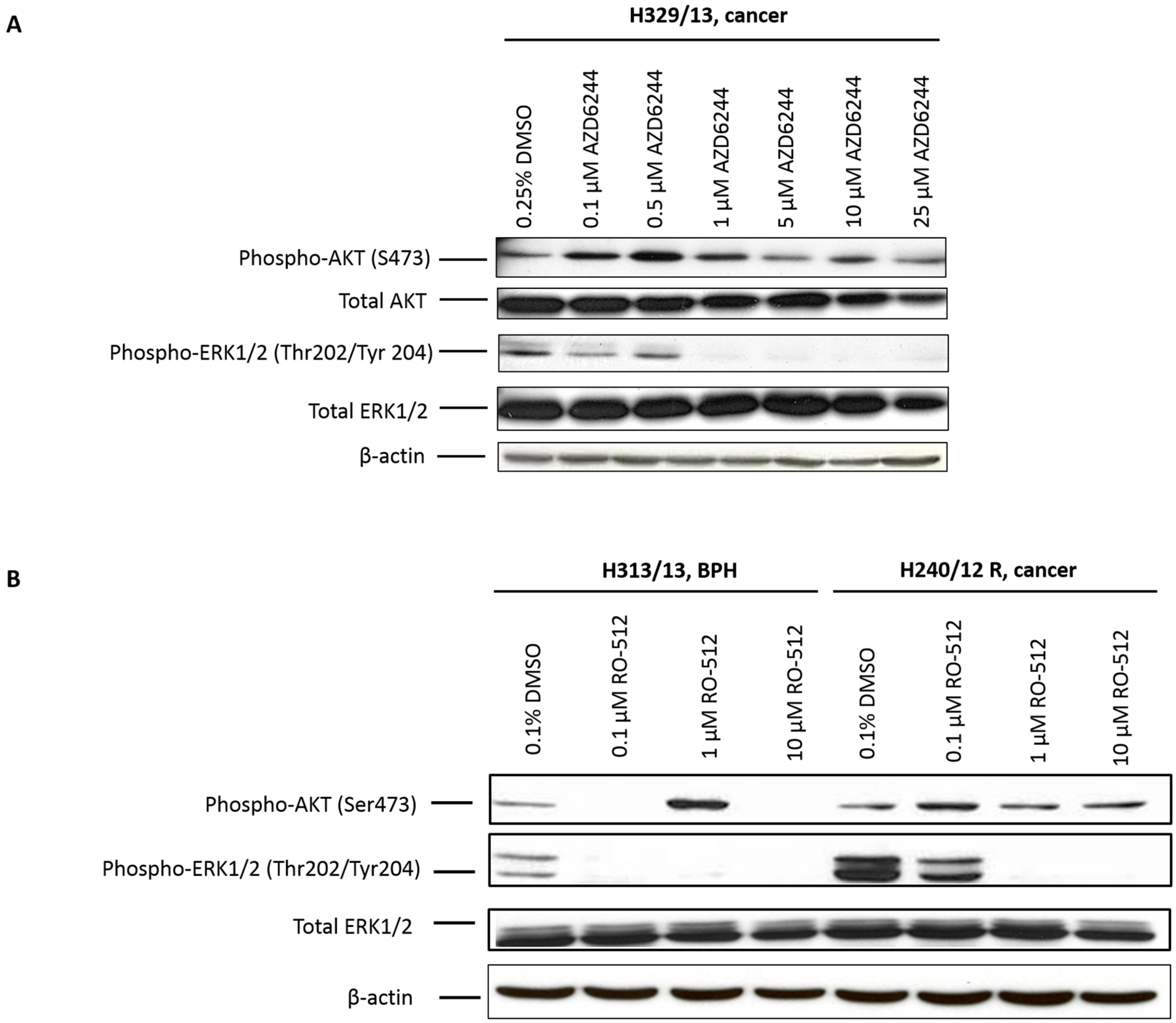

Supplementary Figure 4: Decrease in phospho-ERK1/2 levels following inhibition of MEK1/2. Primary cancer cells H329/13 (GL7) **(A)**, and BPH H313/13 and cancer H240/12 (GL8) cells **(B)**, were treated with increasing concentrations of MEK1/2 inhibitors (0.1 - 25µM of AZD6244) (**A**) and (0.1 - 10 µM of RO-512) (**B**) for 72 hours. Whole cell lysates were prepared and 20 µg of protein was loaded per lane onto a 10% SDS gel, electrotransferred onto PVDF membranes and stained for the biomarkers (phospho-AKT, total AKT, phospho-ERK1/2 and total ERK1/2). Vehicle control cells were treated with 0.25% (**A**) and 0.1% (**B**) DMSO. Staining with β-actin antibody was used as a loading control.
